# The landscape and clinical impact of tumor-associated macrophages and PD-L1 in primary breast cancers and their brain metastases

**DOI:** 10.3389/fimmu.2025.1598293

**Published:** 2025-05-29

**Authors:** Yannik Nicola Zimmer, Benjamin Hanke, Belal Neyazi, Ali Rashidi, Anna Schaufler, Claudia Alexandra Dumitru, Atanas Ignatov, Christian Mawrin, I. Erol Sandalcioglu, Klaus-Peter Stein

**Affiliations:** ^1^ Department of Neurosurgery, Otto-von-Guericke University, Magdeburg, Germany; ^2^ Institute of Pathology, Medizinisches Versorgungszentrum (MVZ) Städtisches Klinikum Dessau, Magdeburg, Germany; ^3^ Department of Gynecology, Otto-von-Guericke University, Magdeburg, Germany; ^4^ Institute of Neuropathology, Otto-von-Guericke University, Magdeburg, Germany

**Keywords:** TAMs, tumor-associated macrophages, breast cancer, brain metastases, CD68, CD86, CD163, PD-L1

## Abstract

**Background:**

Tumor-associated macrophages (TAMs) influence the tumor microenvironment and can contribute to tumor progression. They can polarize into M1 (classically activated) or M2 (alternatively activated) phenotype, which exhibit divergent functional characteristics. The comparison of TAMs between primary breast cancer (BC) and corresponding brain metastases (BMs) remains insufficiently explored and is the focus of this study.

**Methods:**

This study aimed to compare the infiltration of TAMs and PD-L1 expression in primary breast cancer and their brain metastases, by analyzing 27 paired samples and 26 additional brain metastases. Immunohistochemical staining was performed for the following markers: CD68, CD86 (M1), CD163 (M2), and PD-L1.

**Results:**

CD68 showed significantly higher expression levels in brain metastases compared to the corresponding primary breast cancers. In contrast, the expression of CD86 and CD163 showed comparable results between the primary tumors and their brain metastatic counterparts. Macrophages were consistently found to be more frequently present in the tumor stroma compared to the tumor nest. Survival analysis of the primary revealed that high expression of CD163 was associated with a recurrence-free survival. (RFS). Conversely, high expression of CD86 in brain metastases was associated with longer overall survival. Low expression of CD68 and CD163 in brain metastases correlated with the presence of meningeal carcinomatosis. The expression of PD-L1 in the primary tumor did not necessarily reflect the status of PD-L1 in the corresponding brain metastases.

**Conclusions:**

Overall, this study highlights the complex influence of TAMs on the course of primary breast cancers and their brain metastases. The discordant expression of the immune checkpoint molecule PD-L1 underscores the importance of evaluating the PD-L1 status in cerebral metastases to guide potential immunotherapeutic approaches.

## 1 Introduction

Breast cancer has become the most commonly diagnosed malignancy worldwide, with a recorded incidence of 2.26 million cases in 2020 (1). With almost 700.000 deaths, breast cancer is the leading cause of cancer-related mortality among the female population globally (1, 2). Brain metastases are a common complication of advanced malignancies and represent the most prevalent form of intracranial tumors (3). Due to the high incidence of breast cancer, it is the second most common malignancy responsible for brain metastases after lung cancer (4). The prognosis for breast cancer patients with brain metastases is generally poor, with a median overall survival of 7.9 months (5).

TAMs play a central role in shaping the tumor microenvironment during tumor progression. They exhibit a remarkable degree of plasticity, polarizing into M1 or M2 phenotypes based on environmental signals. M1 macrophages are pro-inflammatory, producing IL-6, IL-12, and reactive oxygen species to activate immune responses. In contrast, M2 macrophages promote tissue healing, angiogenesis, and immunosuppression, secreting IL-10 and TGF-β. M1 macrophages are characterized by high iNOS activity, while M2 macrophages regulate arginine metabolism (6). They are recruited through the action of various chemotactic factors such as macrophage colony-stimulating factor (M-CSF) and monocyte chemoattractant protein-1 (MCP-1/CCL-2) and comprise up to 50% of the total tumor mass (7–9). TAMs not only play an essential role in the early stages of metastasis through the degradation of the basal membrane by matrix metalloproteinases (MMPs) (10). TAMs are also involved in promoting the “angiogenic switch” through the release of Vascular Endothelial Growth Factor (VEGF), leading tumor transition from limited blood supply to active angiogenesis (11). Overall, TAMs produce a variety of immunomodulatory cytokines such as IL-4, IL-10, and TGF-β, which induce an anti-inflammatory immune response and thereby suppress cytotoxic reactions (6). In this context their role in PD-1 (Programmed Cell Death Protein 1) and PD-L1 (Programmed Death-Ligand-1) interaction as a central mechanism of immune homeostasis is remarkable (12–15). While the blockade of this axis has revolutionized cancer immunotherapy, resistance to PD-1/PD-L1 inhibitors remains a significant challenge (16). TAMs provide various mechanisms to promote and develop this resistance and expression of PD-L1 on TAMs themselves, rather than on tumor cells, seems of central relevance. This highlights the importance of targeting TAMs in overcoming resistance to PD-1/PD-L1 blockade (16, 17). Several clinical and epidemiological studies have identified a strong correlation between TAM infiltration, poor prognosis, and reduced survival in different cancer types. The majority of these studies analyzed general macrophage populations without taking into consideration the distinct subpopulations of macrophages and their divergent functional roles (18, 19). High levels of M2 macrophages have been linked to decreased overall survival, disease-free survival, and recurrence-free survival in breast cancer (20). Other studies have shown that M1 macrophages exert anti-tumor effects in breast cancer (21).

While the influence of TAMs in primary tumors has been previously investigated, the comparison of TAM infiltration and PD-L1 expression in primary breast cancers and their corresponding brain metastases remains widely unexplored (22). In this study, we evaluated the distinct macrophage subpopulations in 27 paired samples of primary breast cancer and their corresponding brain metastases, as well as an additional 26 brain metastases. This was done using CD68 (M1 + M2), CD86 (M1), and CD163 (M2) as markers, along with pro- and anti-inflammatory surface markers, on consecutive histological slides. The purpose of this study was to answer the following questions: (1) are there differences in the expression of the markers between primary tumors and brain metastases? (2) are there differences in expression between the tumor nest and the tumor stroma? (3) do the subtypes of the primary tumor differ in their expression of the markers? (4) how do the markers correlate with clinical parameters? (5) what is the prognostic value of the markers for patient survival outcomes?

## 2 Materials and methods

### 2.1 Study subjects

The study analyzed 27 paired samples of primary breast tumors and brain metastases, as well as an additional 26 brain metastasis samples. Due to the limited availability of tissue samples in some cases and the presence of extensive necrotic areas, it was not possible to assess every marker for both the tumor nest and tumor stroma for all patients while adhering to the strict protocol. Consequently, the sample size may vary depending on the statistical test. All patients were treated at the Department of Neurosurgery, University Hospital Magdeburg between 2008 and 2021. None of the patients in this cohort received immunotherapy with either Atezolizumab or Pembrolizumab prior to the diagnosis and treatment of cerebral metastases. The ethics committee of Otto von Guericke University Magdeburg approved the study (No. 146/2019) and waived the requirement for written informed consent. The key clinical characteristics of the patients with primary breast tumors and brain metastases are summarized in **Tables 1** and **2**, respectively.

**Table 1:** Clinical characteristics of patients with primary breast cancer included in this study.

	TNBC		HR+/HER2-		HER2+	
	Number	%	Number	%	Number	%
**Total**	9	100	9	100	9	100
Age						
≤ 50	3	33,3	3	33,3	4	44,4
≥ 50	6	66,7	6	66,7	5	55,6
Tumor size						
T1 & T2	4	44,4	5	55,6	7	77,8
T3 & T4	5	55,6	3	33,3	1	11,1
n.d.	0	0,0	1	11,1	1	11,1
Node status						
N0	3	33,3	1	11,1	4	44,4
N+	6	66,7	8	88,9	5	55,6
Metastasic status						
M0	6	66,7	5	55,6	6	66,7
M+	3	33,3	4	44,4	3	33,3
Grading						
G1	0	0.0	0	0,0	0	0,0
G2	2	22,2	7	77,8	4	44,4
G3	7	77,8	2	22,2	5	55,6
Histology						
Non Special Type	7	77,8	6	66,7	8	88,9
Lobular carcinoma	1	11,1	3	33,3	0	0,0
Other	1	11,1	0	0,0	1	11,1
Ki-67						
≤ 25%	3	33,3	5	55,6	3	33,3
≥ 25%	6	66,7	4	44,4	6	66,6
BRCA1						
Positive	1	11,1	0	0,0	1	11,1
Negative	8	88,9	9	100	8	88,9
nCTX						
Yes	4	44,4	2	22,2	4	44,4
No	5	55,6	7	77,8	5	55,6
Surgery						
Lumpectomy	4	44,4	3	33,1	5	55,6
Mastectomy	3	33,3	5	55,6	4	44,4
No surgery	2	22,2	1	11,1	0	0,0
RTX						
Yes	7	77,8	8	88,9	8	88,9
No	2	22,2	1	11,1	1	11,1

Patients were divided into the common subgroups, triple negative breast cancer (TNBC), HR+/HER2- and HER2+. HR, hormone receptors; HER2, human epidermal growth factor receptor; BRCA1, Breast cancer Gene 1; nCTX, neoadjuvant chemotherapy; RTX, radiotherapy; n.d., not determinable.

**Table 2:** Clinical characteristics of the patients with brain metastases included in this study.

	TNBC		HR+/HER2-		HER2+		
	Number	%	Number	%	Number	%
**Total**	15	100	19	100	19	100
Age							
≤ 50	3	20	5	26,3	4	21,0
≥ 50	12	80	14	73,7	15	79,0
Brain metastasis							
Synchronus	2	13,3	2	10,5	0	0,0
Metachronus	13	86,7	17	89,5	19	100
Number of BM							
Solitary	10	66,7	8	42,1	11	57,9
Multiple	5	33,3	10	52,6	8	42,1
n.d.			1	5,3		
Dexamethason treatment							
Yes	8	53,3	8	42,1	6	31,6
No	7	46,7	11	57,9	12	63,2
n.d.					1	5,3
Meningeal carcinomatosis							
Yes	2	13,3	3	15,8	3	15,8
No	13	86,7	16	84,2	16	84,2
Relaps/New BM						
Yes	4	26,7	4	21,1	5	26,3
No	11	73,3	15	78,9	14	73,7

BM, brain metastases; n.d., not determinable.

### 2.2 Immunohistochemical staining

The expression of the markers was evaluated in FFPE (formalin-fixed paraffin-embedded) tissues, which were Since the immunotherapeutic agent atezolizumab was first approved for the treatment of patients with metastatic, triple-negative and PD-L1-positive breast cancer in 2019, no patient received immunotherapy prior to the diagnosis and treatment of cerebral metastasis.processed into tissue microarrays (TMAs) according to the standard method established in our laboratory (23, 24). The samples were stained with the following primary antibodies: CD68 (Dako, KP1), CD86 (Cell Signaling, E2G8P), CD163 (Biolegend, QA19A16) and PD-L1 (Dako, 22C3). After staining, the samples were digitalized with an Aperio VERSA high-resolution whole slide scanner and analyzed with the Aperio ImageScope V12.1.0.5029 software (both from Leica Biosystems, Nussloch, Germany). Cells stained with the indicated antibody were calculated per hot-spot region, with three fields per section evaluated at 200× magnification.

### 2.3 Macrophage quantification

**Figure 1:**
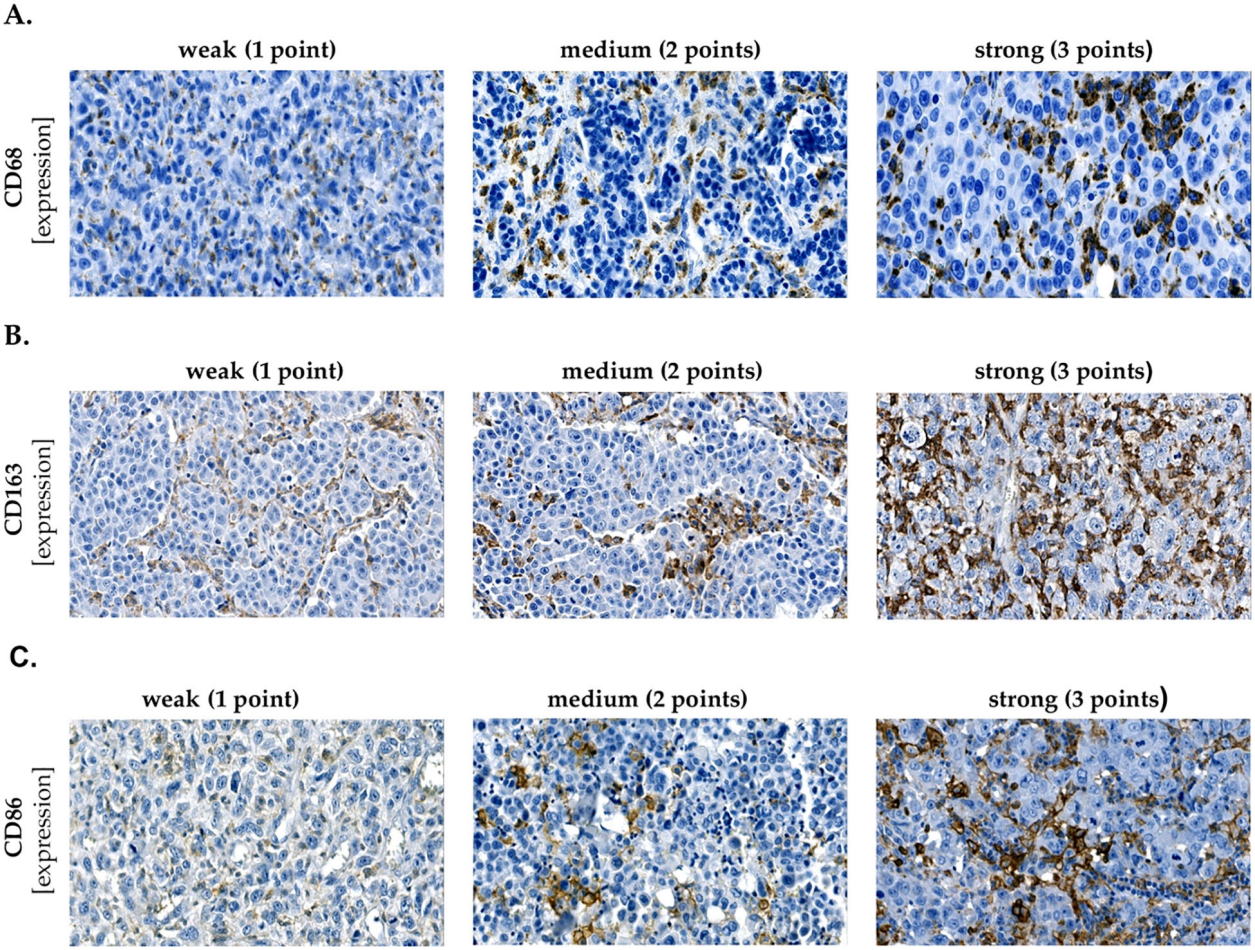
Staining intensity. Representative micrographs showing weak (1 point), medium (2 points) and strong (3 points) expression of **(A)** CD68, **(B)** CD163, **(C)** CD86. The IRS was subsequently calculated according to the formula: SI (staining intensity) × PP (percentage of positive cells).

Despite strict adherence to the protocols, variability in staining intensity was observed across the samples. To account for this, an Immunoreactive Score (IRS) was used to quantify CD68, CD163, and CD86. This modified IRS approach involves the assessment of both staining intensity and the distribution of positive cells within the tissue samples (25, 26). Briefly, the Immunoreactive Score (IRS) was calculated as SI (staining intensity) × PP (percentage of positive cells). SI was assigned as follows: 1 = weak, 2 = moderate, 3 = strong (**Figure 1**). PP was defined as: 1 = 0–10%, 2 = 10–25%, 3 = 26–40%, 4 = >40%. Patients were then dichotomized into two groups based on the median IRS score of CD68, CD86, and CD163 expression: high expression group (> median score) and low expression group (≤ median score). Authors Y.N.Z. and K-P.S. performed blinded histological scoring independently.

### 2.4 PD-L1 quantification

The evaluation of PD-L1 expression was carried out by an experienced pathologist B.H. In clinical practice, the following three scores have become established: Tumor Proportion Score (TPS), Immune Cell Score (IC), and Combined Positive Score (CPS) (27). In line with the KEYNOTE-522 approval study for the antibody Pembrolizumab for the treatment of TNBC, the CPS was used. We decided to use a cut-off value of 1 to better understand the impact of PD-L1 on survival times and correlations with clinical characteristics (28). This method considers the expression in both tumor and immune cells. When counting positive cells, only those with membrane-bound expression were included.

### 2.5 Statistical analysis

Statistical analysis was performed using the software program R (version 4.3.0) in combination with RStudio (version 2023.06.01). Kaplan-Meier curves were generated using GraphPad Prism 10. Differences in marker expression between tumor nest and tumor stroma and between primary tumors and brain metastases were analyzed using the Wilcoxon test. Survival curves were constructed using the Kaplan-Meier method, and statistical significance was assessed by univariate analysis using the log-rank test. Clinical associations were assessed using Fisher’s exact test. The statistical significance was set at p ≤ 0.05.

## 3 Results

### 3.1 Comparison of TAM distribution between the tumor nest and tumor stroma

The expression patterns of CD68, CD86, and CD163 were first analyzed to assess differences between the tumor nest and tumor stroma. This analysis was conducted on the overall cohort, followed by subgroup analyses of triple-negative, HR+/HER2-, and HER2+ tumors. Statistical significance was assessed using Wilcoxon tests, and the results are presented in **Table 3**. The Wilcoxon test revealed a significant difference in the expression of the markers CD68, CD86, and CD163 between the tumor nest and tumor stroma in both the primary tumor and brain metastases across the overall cohort (p < 0.0001). For CD68 expression in primary tumors, a significant difference between the tumor nest and tumor stroma was observed exclusively in HER2+ tumors (p = 0.008). For CD86, significant differences were found in the triple-negative (p = 0.016) and HER2+ (p = 0.016) subgroups of the primary tumor. CD163 showed a significant difference in all subgroups of the primary tumors.

**Table 3:** Results of the Wilcoxon test for expression differences between TN and TS in primary breast cancer (BC) and brain metastases (BM) respectively.

		BC				BM			
		n	Median		p	n	Median		p
			TN	TS			TN	TS	
**CD68**	Total	26	2	4	< 0.0001	44	3	8	< 0.0001
	TNBC	9	2	4	0.125	12	4	9	0.008
	HR+/HER2 -	8	2	4	0.094	15	2	8	0.0001
	HER2 +	9	2	4	0.008	17	3	6	< 0.0001
**CD163**	Total	26	2	6	< 0.0001	44	2	6	< 0.0001
	TNBC	9	2	4	0.016	12	2	8	0.001
	HR+/HER2 -	8	1	2	0.016	15	1	4	0.0001
	HER2 +	9	2	6	0.004	17	2	6	< 0.0001
**CD86**	Total	26	1	4	< 0.0001	44	2	6	< 0.0001
	TNBC	9	1	4	0.016	12	1	6	0.002
	HR+/HER2 -	8	1	2	0.125	15	2	4	0.033
	HER2 +	9	2	4	0.016	17	2	6	< 0.0001

TN, tumor nest; TS, tumor stroma.

### 3.2 Comparison of TAMs between primary breast cancer and brain metastases

Next, we compared the expression of 27 primary breast cancers and their paired brain metastases for CD68, CD163 and CD86. Statistical significance was determined using Wilcoxon tests, with the results presented in **Table 4**. CD68 expression was significantly higher in the tumor stroma of metastases compared to the corresponding primary tumors (p = 0.011), with this difference primarily driven by HR+/HER2- tumors (p = 0.016). Additionally, subgroup analysis revealed a significantly higher CD86 expression in the tumor nests of metastases from HR+/HER2- tumors (p = 0.031). As noted in Section 2.1, the tumor stroma could not be evaluated in all samples.

**Table 4:** Results of the Wilcoxon test for the expression differences between TN and TS. Comparison of BC and BM.

		TN				TS			
		n	Median		p	n	Median		p
			BC	BM			BC	BM	
**CD68**	Total	27	2	3	0.094	21	4	6	0.011
	TNBC	9	2	4	0.344	7	6	9	0.25
	HR+/HER2 -	9	2	2	0.281	5	6	6	0.75
	HER2 +	9	2	3	0.375	9	4	6	0.016
**CD163**	Total	27	2	2	0.548	21	6	6	0.576
	TNBC	9	2	2	1	7	6	9	0.75
	HR+/HER2 -	9	1	1	0.375	5	3	6	0.75
	HER2 +	9	2	2	0.688	9	6	6	0.844
**CD86**	Total	27	1	2	0.104	21	4	6	0.377
	TNBC	9	1	2	0.375	7	6	5	0.625
	HR+/HER2 -	9	1	3	0.031	5	3	4	0.5
	HER2 +	9	2	2	0.797	9	4	8	0.5

### 3.3 Comparison of PD-L1 expression between primary breast cancer and brain metastases

**Figure 2:**
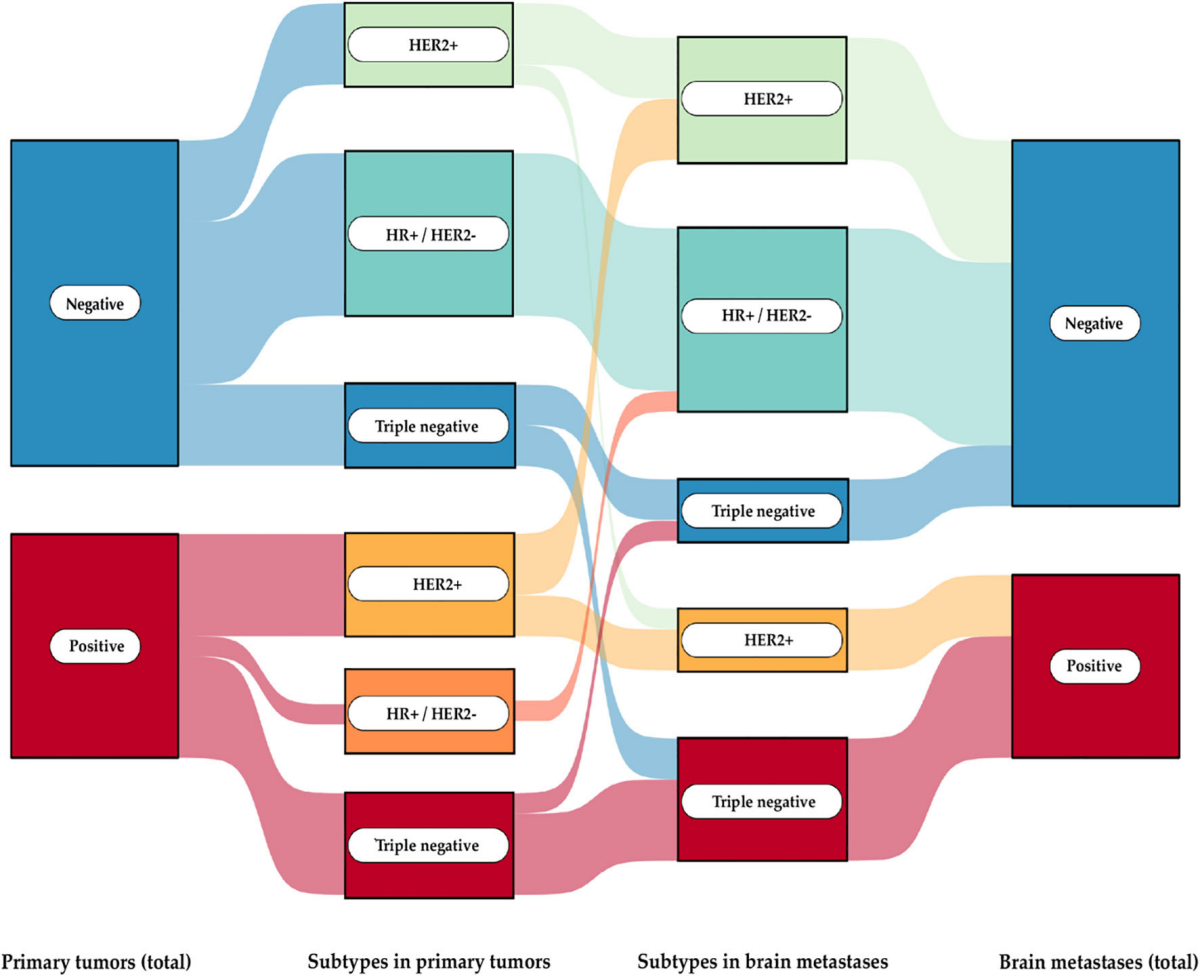
Sankey diagram of PD-L1 expression in primary breast cancer and their brain metastases The flow of PD-L1 expression status (positive or negative) across different stages and subtypes of breast cancer progression: from primary tumors to subtypes in both primary tumors and brain metastases, culminating in the PD-L1 expression status in brain metastases.

For PD-L1 status, we found that the status of the primary tumor does not necessarily predict the status of the brain metastasis (**Figure 2**). Overall, PD-L1 expression was observed in 40.7% of the primary BCs and in 33.3% of their corresponding BMs (CPS > 1). Discordant PD-L1 expression was noted in 7 out of 27 cases (25.9%): 4 cases showed positive expression in the primary tumor but negative expression in the corresponding brain metastasis, while 3 cases exhibited no PD-L1 expression in the primary tumor but positive expression in the brain metastasis. Closer analysis revealed that positive PD-L1 expression was particularly associated with the triple-negative and HER2+ subgroups. In the HR+/HER2- group, PD-L1 expression was positive in only one case. However, the corresponding brain metastasis showed a negative PD-L1 status. When examining all 53 brain metastases, positive PD-L1 expression was detected in 22 samples. Subgroup analysis showed the following positive expression rates: TNBC (66,66%), HR+/HER2- (21,05%), and HER2+ (42,10%)

**Figure 3:**
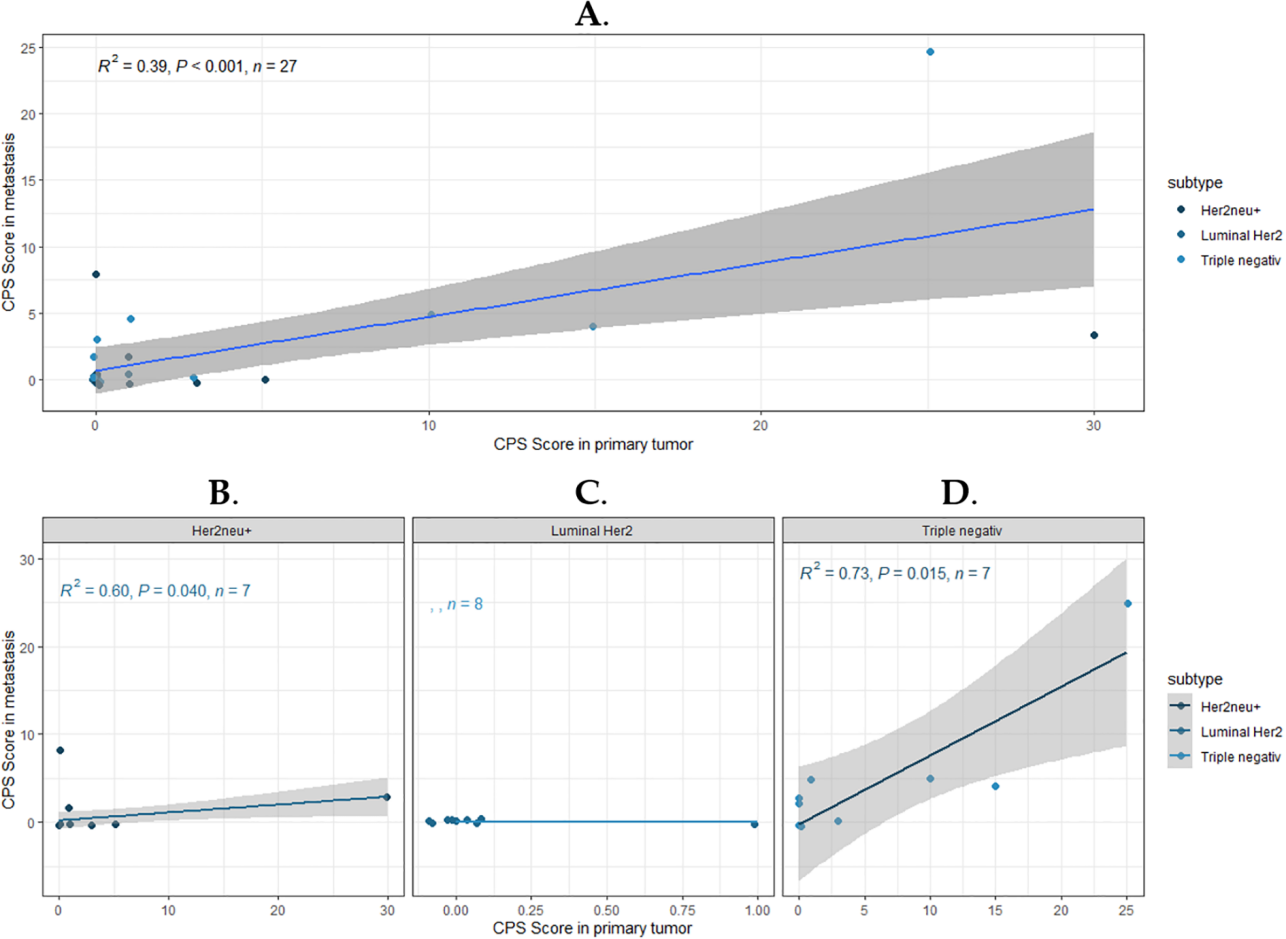
Correlation of PD-L1 expression between primary breast cancer and brain metastasis. The graphs show scatter plots and corresponding regression lines to illustrate a linear relationship. **(A)** represents the correlation for the entire cohort. **(B–D)** show the subgroups: HER2 +, HR+/HER2 -, and Triple-negative. The strength of the correlation is represented by the coefficient of determination (R²), which is displayed along with the p-value and sample size (n) in the upper left corner of the plots.

A regression analysis was subsequently performed to assess the impact of PD-L1 expression in the primary tumor on the status of brain metastasis. The CPS in the primary tumor was considered the independent variable (x), and the CPS in the metastasis was considered the dependent variable (y). Initially, the entire cohort was analyzed, followed by subgroup analyses, with the results presented in **Figure 3**.

The coefficient of determination (R²) for the entire cohort indicates that approximately 39% of the variation in the CPS of the metastasis can be explained by the variation in the CPS of the primary tumor (**Figure 3A**). This suggests that the regression line does not fully account for all fluctuations in the CPS of the metastasis, and the CPS in the metastasis is not necessarily determined by the CPS of the primary tumor. The significance level (p < 0.001) indicates that the relationship between the CPS in the primary tumor and the metastasis is statistically significant. When examining the subgroups separately, it becomes apparent that this correlation is primarily observed in the PD-L1 expression of TNBC (R² = 0.73; p = 0.003) and HER2/neu+ tumors (R² = 0.03; p = 0.675) (**Figures 3B–D**). PD-L1 expression is generally scarcely observed in Luminal HER2/neu- type patients, which is why no calculation could be performed (**Figure 3C**).

### 3.4 Impact of TAMs and PD-L1 on recurrence-free survival (primary-BM)

**Figure 4:**
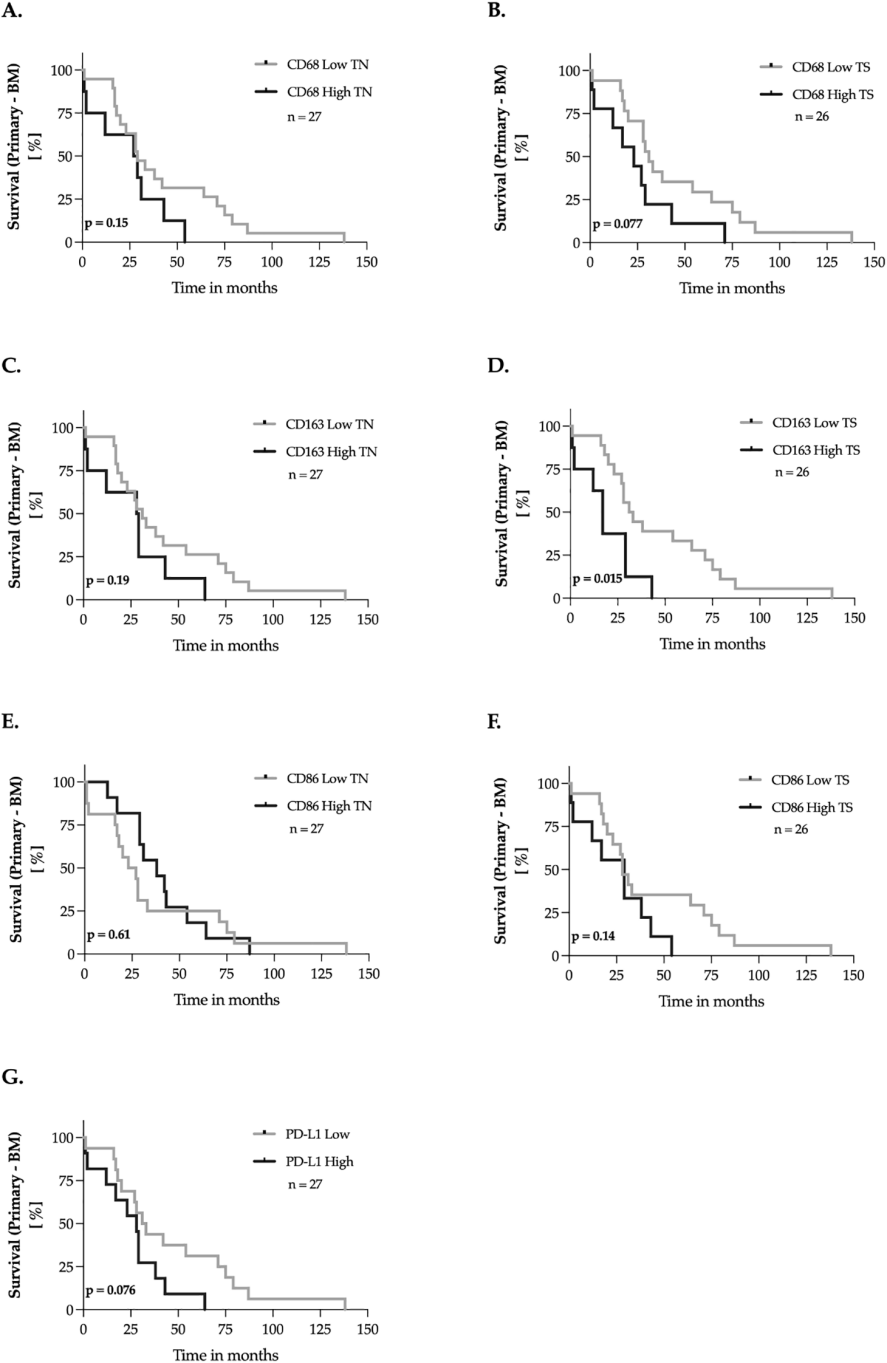
Marker expression and the recurrence-free survival of breast cancer patients – univariate analysis. **(A–G)** The expression levels of the markers were dichotomized into ‘low’ and ‘high’ according to the median-split method. Kaplan-Meier curves were generated for the 150-months recurrence-free survival and statistical analysis was performed with the log-rank test. The *p-*values are indicated in the lower- left corner of each plot. Sample sizes are indicated in the upper-right corner of each plot. TN, tumor nest; TS, tumor stroma.

Next, we analyzed the impact of CD68, CD163, CD86, and PD-L1 expression on recurrence-free survival (RFS), defined as the time between the initial diagnosis of the primary breast tumor and cerebral progression. Expression levels of the markers were dichotomized into “low” and “high” groups using the median-split method. Kaplan-Meier survival curves were then generated for each marker, and univariate survival analyses were performed using the log-rank test (**Figure 4**). We found that the high expression of CD163-positive macrophages in the tumor stroma, was associated with an earlier occurrence of brain metastases (p = 0.015, **Figure 4D**). For the remaining markers, no significant impact on recurrence-free survival was observed.

### 3.5 Impact of TAMs and PD-L1 on overall survival (primary-death)

**Figure 5:**
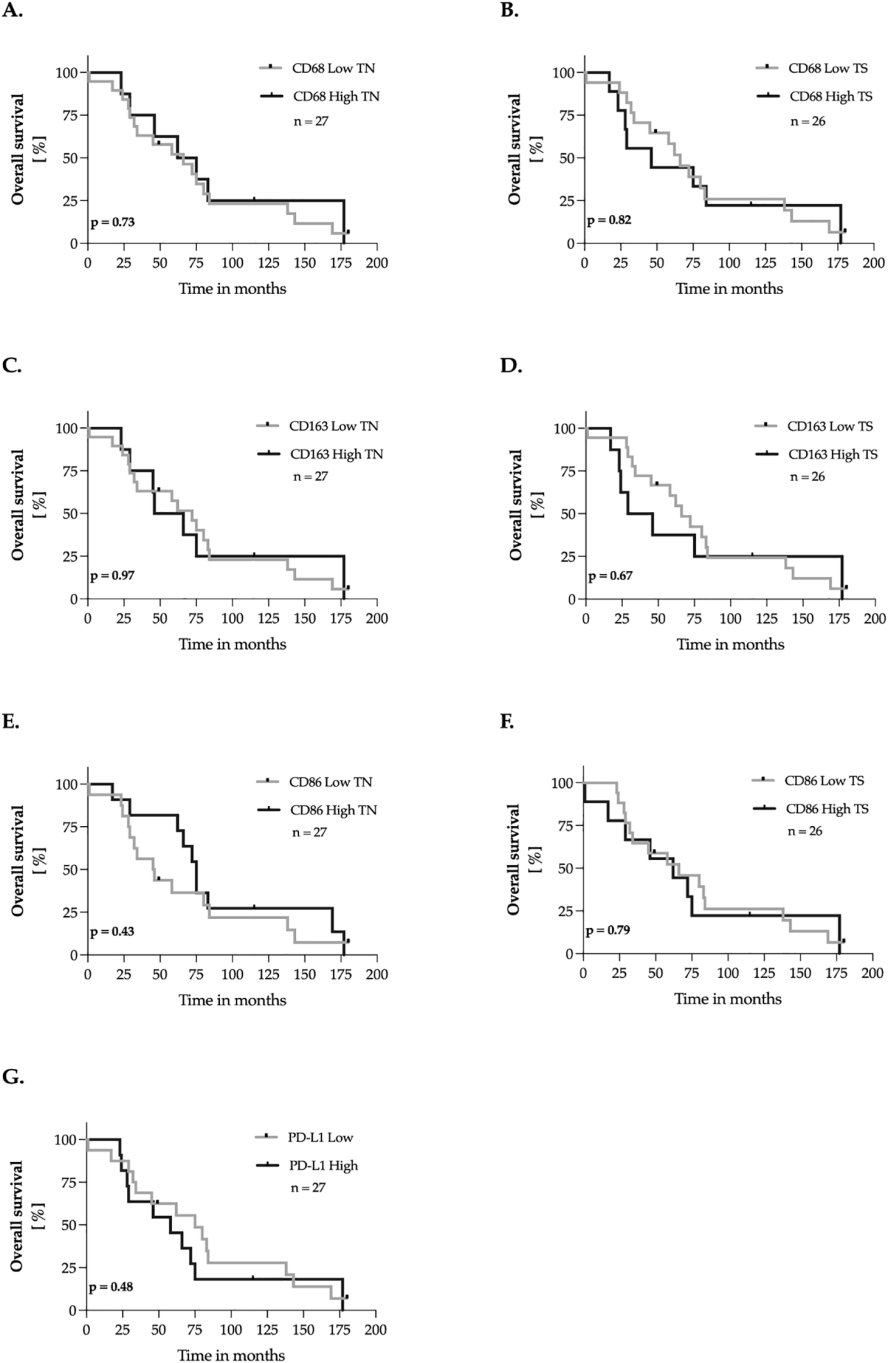
Marker expression and overall survival of breast cancer patients – univariate analysis. **(A–G)** The expression levels of the markers were dichotomized into ‘low’ and ‘high’ according to the median-split method. Kaplan-Meier curves were generated for overall survival and statistical analysis was performed with the log-rank test. The *p-*values are indicated in the lower- left corner of each plot. Sample sizes are indicated in the upper-right corner of each plot. TN, tumor nest; TS, tumor stroma.

Subsequently, we evaluated the impact of CD68, CD163, CD86, and PD-L1 expression in primary tumor tissues on overall survival, defined as the time from the initial breast cancer diagnosis to death. The methodology applied adhered to the approach outlined in Section 3.4. No significant association was observed between CD68, CD163, CD86, and PD-L1 expression in the tumor nest or tumor stroma and patients´ overall survival (**Figure 5**).

### 3.6 Impact of TAMs and PD-L1 on brain metastasis survival (BM-death)

**Figure 6:**
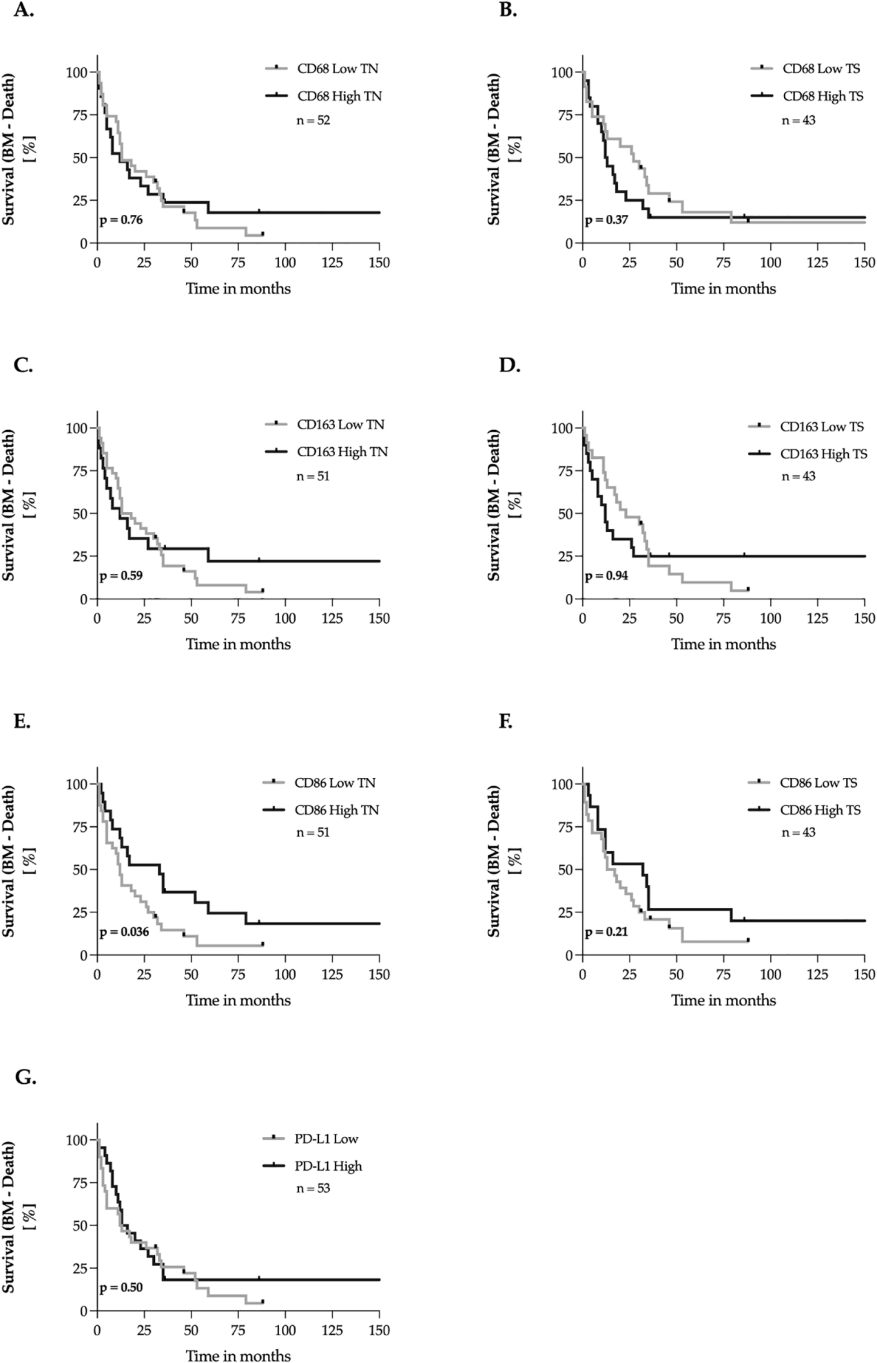
Marker expression and brain metastasis survival (BM – Death) – univariate analysis. **(A–G)** The expression levels of the markers were dichotomized into ‘low’ and ‘high’ according to the median-split method. Kaplan-Meier curves were generated for overall survival and statistical analysis was performed with the log-rank test. The *p-*values are indicated in the lower- left corner of each plot. Sample sizes are indicated in the upper-right corner of each plot. TN, tumor nest; TS, tumor stroma.

We investigated the impact of CD68, CD163, CD86, and PD-L1 expression in brain metastasis tissues on the interval between the initial diagnosis of brain metastases and death. This interval is a key clinical parameter, providing insights into metastasis-specific survival, disease progression, and therapeutic efficacy. The methodology followed the procedures outlined in Sections 3.4 and 3.5 (**Figure 6**). Our analysis revealed a significantly longer brain metastasis survival (BMS) in patients with high CD86 expression in the tumor nest of brain metastases, showing a 63.6% increase in median survival (p = 0.036, **Figure 6E**). In contrast, CD86 expression in the tumor stroma had no measurable impact on this interval. Additionally, no significant association was found between CD68, CD163, or PD-L1 expression in either the tumor nest or tumor stroma and BMS.

### 3.7 TAMs/PD-L1 and clinicopathological features of primary breast cancer

Next, we investigated the association between CD68, CD163, CD86, and PD-L1 expression and the clinicopathological features of primary breast tumors (**Supplementary Tables 1A-D**). Fisher’s exact test was performed to analyze the following characteristics: TNM stage, grading, hormone receptor status, HER2 status, and the impact of neoadjuvant chemotherapy. A significant association was found between high CD163 expression in the tumor stroma of the primary tumor and positive hormone receptor status (p = 0.038, **Supplementary Table 1B**). Additionally, high CD86 expression in the tumor nest was associated with high-grade tumors (p = 0.018, **Supplementary Table 1C**), while no significant associations were observed for CD68 and PD-L1.

### 3.8 TAMs/PD-L1 and clinicopathological features of brain metastases

Finally, we analyzed the association between CD68, CD163, CD86, and PD-L1 expression and the clinicopathological features of brain metastases (**Supplementary Tables 2A-D**). Using Fisher’s exact test, we examined the following characteristics: meningeal carcinomatosis, dexamethasone treatment, number of brain metastases (solitary vs. multiple), presentation (synchronous vs. metachronous), and cerebral relapse. We found a significant association between low CD68 expression in the tumor nest of brain metastases and the occurrence of meningeal carcinomatosis (p = 0.016, **Supplementary Table 2A**). Similarly, polarization analysis revealed a significant association between low CD163 expression in the tumor nest of brain metastases and the presence of meningeal carcinomatosis (p = 0.04, **Supplementary Table 2B**). No significant associations were observed for CD86 and PD-L1.

## 4 Discussion

### 4.1 TAMs and PD-L1 expression in primary breast cancer and paired brain metastases

The brain has long been considered immune-privileged due to the blood-brain barrier (BBB). However, recent research suggests that the brain is not immune-privileged but rather immunologically unique, particularly following the discovery of functional lymphatic vessels within the central nervous system. Especially in brain tumors, immune cell infiltration is a common phenomenon (29). Following damage to the BBB, peripheral monocytes can significantly contribute to the macrophage pool within the central nervous system (30, 31). Bowman et al. demonstrated that, in addition to microglia, infiltrating bone marrow-derived macrophages are present in cerebral metastases (32). We likewise found a broad infiltration of CD68 expressing cells in the tumor stroma in the overall cohort, with significantly higher levels in metastases compared to corresponding primary tumors. This difference was particularly pronounced in HER2-positive tumors. Further subgroup analysis revealed higher CD86 expression in the cerebral metastases of HR+/HER2- tumors. In contrast, we found no disparities in the expression of the markers CD68, CD163, and CD86 within the tumor nest across the entire cohort. These results emphasize the need to differentiate between individual tumor regions and cell polarization. According to this work, several studies have shown that tumor-associated macrophages are present in both the tumor nest and the tumor stroma (20, 33). The findings of Mahmoud et al. demonstrate that the majority of TAMs are located in the stroma, a conclusion that aligns with the results of our study (20). We only identified one study by Liu and co-workers investigating the expression differences of macrophage markers between primary tumors and brain metastases. The analysis included 17 paired samples of non-small cell lung carcinomas and brain metastases as well as 45 unpaired brain metastases. No significant differences in the expression of CD68 and CD163 were found. Their macrophage quantification differed from our approach, as they did not distinguish between the tumor nest and stroma, which needs to be emphasized. In addition, the examined primary tumors differ from each other (34).

Of utmost relevance, the PD-L1 status of the primary tumor did not necessarily predict the status of its brain metastases in our cohort (**Figure 2**). So far, PD-L1 expression is not routinely assessed in neuropathological diagnostics for brain metastases of breast cancer and this prompts further inquiry into the potential value of routine PD-L1 testing in brain metastases. Similar discordant PD-L1 expression patterns between breast tumors and brain metastases were reported recently (35). Until know, there is an absence of a non-invasive method capable of predicting the response of brain metastases to immunotherapy. In 2023, Brastianos et al. conducted the first study evaluating the response of brain metastases to pembrolizumab, regardless of their underlying PD-L1 status. Patients were grouped by tumor type, with the largest cohort consisting of breast cancer patients (n = 35). The primary endpoints included complete response, partial response, and stable disease. The analysis revealed that 42.1% of patients exhibited a positive response to the therapy, with a response rate of 37.0% observed among breast cancer patients. Of particular interest is the observation that no significant disparities were detected among the various breast cancer subtypes. However, only 8.8% of the total cohort achieved complete or partial remission (36). Tiezzi et al. proposed CD86 as a potential prognostic marker for predicting the efficacy of immunotherapy in breast cancer, particularly in triple-negative subtypes (37). While immunotherapy has demonstrated efficacy in treating brain metastases across various tumor types, not all patients respond to this treatment. Consequently, further research is imperative to elucidate the underlying mechanisms and enhance the predictive capacity of therapeutic interventions (36).

### 4.2 Impact of TAMs and PD-L1 in primary tumor and brain metastases on survival

Shorter survival times such as OS and BMS or RFS are often associated with aggressive tumor biology and rapid disease progression. In the present study, a significant correlation was found between a high expression of CD163 in the primary tumor stroma and a shorter RFS (**Figure 4D**). Consistent with our findings, a high incidence of CD163-positive macrophages in breast cancer was associated with earlier progression in several studies (22, 38). In contrast to the results of Tiainen et al., we did not find an impact of CD163 and CD68 on overall survival (22). In this work, consistent with the results of a previous study on breast cancer, we were unable to demonstrate an association between CD86 and RFS or OS (39). In contrast we found a significantly longer BMS with high CD86 expression in the tumor nest of the cerebral metastases in our cohort (**Figure 6E**). Similar favorable prognoses have also been reported for M1 macrophages in primary tumors of melanomas and lung carcinomas, but not in their brain metastases (40, 41). Although M1 macrophages are thought to have antitumor properties, there are also contrary findings in the literature: In one study, CD86 was associated with a poorer prognosis in multivariate analysis (37). In contrast, a study on HER2-positive breast cancer showed that a high density of M1 macrophages labeled with iNOS correlated with longer overall survival (21).

We did not find a significant association between PD-L1 and RFS or OS. In a study by Qin et al, 870 breast cancer cases were analyzed for PD-L1 expression. They found that high PD-L1 expression correlated with significantly shorter recurrence-free survival and overall survival (42). A meta-analysis of 2546 cases confirmed this finding and showed that overall survival is shortened in breast cancer with high PD-L1 expression (43). In the pivotal KEYNOTE 522 trial of the PD-1 antibody pembrolizumab for the treatment of TNBC, the combination of pembrolizumab and chemotherapy, followed by pembrolizumab alone, resulted in a 37% reduction in the risk of disease progression or death. Patients must have positive PD-L1 expression with a CPS ≥ 10 to be eligible for this therapy (28).

### 4.3 Associations of TAMs and PD-L1 with clinicopathological features of primary breast cancer

We observed a significant association between high expression of the M2 macrophage marker CD163 in the tumor stroma and negative hormone receptors, which is in accordance with previous findings (**Supplementary Table 1B**) (44). Others have also demonstrated a correlation between CD163-positive macrophages and higher T-stage, lymph node metastases, increased grade, elevated Ki-67 proliferation index, and HER2 positivity (22, 44). Furthermore, CD86 in the tumor nest correlated with higher tumor grading, indicating unfavorable properties of CD86 (**Supplementary Table 1C**). One study group intensively investigated the role of M1 macrophages, using transcriptome analysis, and identified both positive and negative properties. In contrast to previous studies, they found no correlation between M1 macrophages and improved response to neoadjuvant chemotherapy or longer survival. Defying expectations, there was a significant correlation with clinically aggressive tumor characteristics, such as a higher Nottingham grade and increased Ki-67 proliferation index in breast carcinomas. They postulated that tumors with high M1 content exhibit increased immune activity to compensate for tumor aggressiveness. In addition to the expected cytokines such as IFN-γ, the release of pro-tumor TGF-β was also detected. Tumors with many M1 macrophages also showed a strong infiltration of both favorable and unfavorable immune cells. M1 macrophages had a significantly higher cytological activity value (CYT), which could indicate a favorable tumor microenvironment. The CYT is composed of the expression of perforin and granzyme, which are secreted by cytotoxic T cells (39). High CYT levels have been associated with longer survival in previous studies (45).

### 4.4 TAMs and meningeal carcinomatosis

Meningeal carcinomatosis occurs in approximately 3–8% of all cancer cases and represents an advanced tumor stage as well as a serious complication. In this process, the tumor cells enter into the subarachnoid space and spread via the cerebrospinal fluid (CSF), finally settling in areas with reduced CSF flow, such as the cauda equina, the hippocampal fissure or the basal cisterns (46). The prognosis is very poor, with a life expectancy of only 4–6 weeks without treatment (47). We found a significant association between low expression of CD68 in the tumor nest of brain metastases and the occurrence of meningeal carcinomatosis (**Supplementary Table 2A**). Polarization analysis also showed a significant correlation with low CD163 expression (**Supplementary Table 2B**). Contrary to our expectation, these results suggest that low expression of TAMs in the brain metastasis is associated with a higher risk of this fatal event. There is evidence, that meningeal carcinomatosis is accompanied by higher levels of macrophages in CSF samples. Vice versa, a high number of macrophages in CSF samples in patients with diverse solid tumors correlates with the presence of atypical or malignant cells. Using a cut-off value for the number of macrophages, Kobayshi et al. were able to cytologically distinguish tumor-positive from tumor-negative CSF samples (48). The underlying pathophysiology is not yet fully elucidated, and further research is necessary to address this. A comparison of the TAMs between the brain metastasis and the corresponding CSF could provide valuable insights.

### 4.5 Limitations of the study

We are aware of several limitations of this study. Due to small sample size the statistical results should be interpreted with caution. A further problem was posed by the tissue samples themselves, some of which contained only slightly vital tumor cells or showed bleeding and large areas of necrosis. Therefore, it was not possible to determine an IRS for the tumor nest and tumor stroma in every sample while adhering to the strict methodology. Furthermore, the quantification of TAMs is generally challenging, as there is no standardized and established method. Various approaches are used, including cell counting, semi-quantitative evaluation, the use of IRS and area calculations using Fiji/ImageJ (49–51). In addition, the analysis is performed either separately for tumor nest and tumor stroma or exclusively for a specific region (20, 49). This variety of methods reflects the complexity and inconsistent quantification of TAMs. Finally, the high plasticity of macrophages needs to be stressed. Therefore, the term “M1-like” and “M2-like” macrophages is more and more used, as a simple dichotomization of polarization might no longer be appropriate (52). Finally, it must be emphasized that macrophages represent one player among a multitude of immune cells in shaping tumor microenvironment. Especially mutual cell interactions are not considered in our study, such as TAM CD86 and CD163 expression and its role for CD4+ and CD8+ T cell function and response (53). As these cell-cell interactions may be the target of further treatment approaches, it is worth focusing future studies on this aspect.

## 5 Conclusion

The aim of this study was to compare the expression of CD68, CD86, CD163 and PD-L1 in breast carcinomas and their paired brain metastases. Our results show a higher expression of CD68 in the tumor stroma of brain metastases, whereas CD86 and CD163 showed comparable levels in both locations. For PD-L1, we observed that its status in the primary does not necessarily reflect its expression in brain metastases, highlighting the importance of specific PD-L1 testing in brain metastases. High CD163 expression in the tumor stroma of the primary was associated with RFS, while high CD86 expression in the tumor nest of brain metastases correlated with longer brain metastasis-specific survival. In addition, CD163 was associated with hormone receptor-negative breast cancer, while CD86 correlated with higher tumor grade. In brain metastases, a significant association was found between low expression of CD68 and CD163 and the presence of carcinomatous meningitis. Overall, these findings contribute to a better understanding of the pathophysiology of tumor-associated macrophages (TAMs) and PD-L1 and may help to identify potential targets for improved therapeutic strategies in breast cancer and brain metastases.

## Data Availability

The raw data supporting the conclusions of this article will be made available by the authors, without undue reservation.
