# Supplementary material for: The landscape and clinical impact of tumor-associated macrophages and PD-L1 in primary breast cancers and their brain metastases
Source: Front Immunol. 2025 May 29;16:1598293. doi: 10.3389/fimmu.2025.1598293 (PMC12159042; doi:10.3389/fimmu.2025.1598293)
Supplement: Supplementary file 1 [file DataSheet1.docx]

**Supplement**

**Supplement 1A**: Results of the Fisher´s exact test for association between clinicopathological features of primary breast cancer and expression of CD68. TN: tumor nest; TS: tumor stroma; nCTX: neoadjuvant chemotherapy; n.d.: not determinable.

|  | **CD68, TN** | | | **CD68, TS** | | |
| --- | --- | --- | --- | --- | --- | --- |
|  | **High,**  **n = 8** | **Low,**  **n = 19** | **p** | **High,**  **n = 9** | **Low,**  **n = 17** | **p** |
| **Tumor size** |  |  | 0,8 |  |  | 0,12 |
| 1 | 2 | 5 |  | 2 | 5 |  |
| 2 | 2 | 7 |  | 1 | 8 |  |
| 3 | 2 | 2 |  | 2 | 2 |  |
| 4 | 1 | 4 |  | 3 | 1 |  |
| n.d. | 1 | 1 |  | 1 | 1 |  |
| **Node status** |  |  | >0.9 |  |  | 0,7 |
| N0 | 2 | 6 |  | 2 | 6 |  |
| N+ | 6 | 13 |  | 7 | 11 |  |
| **Metastasic status** |  |  | 0,7 |  |  | 0,7 |
| M0 | 6 | 11 |  | 5 | 12 |  |
| M+ | 2 | 8 |  | 4 | 5 |  |
| **Grading** |  |  | 0,7 |  |  | >0.9 |
| 2 | 3 | 10 |  | 4 | 9 |  |
| 3 | 5 | 9 |  | 5 | 8 |  |
| **Estrogen receptor** |  |  | 0,7 |  |  | 0,4 |
| Positive | 4 | 12 |  | 4 | 11 |  |
| Negative | 4 | 7 |  | 5 | 6 |  |
| **Progesteron receptor** |  |  | 0,7 |  |  | 0,4 |
| Positiv | 4 | 12 |  | 4 | 11 |  |
| Negativ | 4 | 7 |  | 5 | 6 |  |
| **HER2/neu** |  |  | 0,2 |  |  | 0,4 |
| Positive | 1 | 8 |  | 2 | 7 |  |
| Negative | 7 | 11 |  | 7 | 10 |  |
| **nCTX** |  |  | >0.9 |  |  | >0.9 |
| Yes | 3 | 7 |  | 3 | 6 |  |
| No | 5 | 12 |  | 6 | 11 |  |

**Supplement 1B**: Results of the Fisher´s exact test for association between clinicopathological features of primary breast cancer and expression of CD163. TN: tumor nest; TS: tumor stroma; nCTX: neoadjuvant chemotherapy; n.d.: not determinable.

|  | **CD163, TN** | | | **CD163, TS** | | |
| --- | --- | --- | --- | --- | --- | --- |
|  | **High,**  **n = 8** | **Low,**  **n = 19** | **p** | **High,**  **n = 8** | **Low,**  **n = 18** | **p** |
| **Tumor size** |  |  | >0.9 |  |  | 0,8 |
| 1 | 2 | 5 |  | 1 | 6 |  |
| 2 | 3 | 6 |  | 3 | 6 |  |
| 3 | 1 | 3 |  | 1 | 3 |  |
| 4 | 1 | 4 |  | 2 | 2 |  |
| n.d. | 1 | 1 |  | 1 | 1 |  |
| **Node status** |  |  | 0,4 |  |  | 0,4 |
| N0 | 1 | 7 |  | 1 | 7 |  |
| N+ | 7 | 12 |  | 7 | 11 |  |
| **Metastasic status** |  |  | >0.9 |  |  | 0,4 |
| M0 | 5 | 12 |  | 4 | 13 |  |
| M+ | 3 | 7 |  | 4 | 5 |  |
| **Grading** |  |  | >0.9 |  |  | 0,7 |
| 2 | 4 | 9 |  | 3 | 10 |  |
| 3 | 4 | 10 |  | 5 | 8 |  |
| **Estrogen receptor** |  |  | 0,7 |  |  | **0,038** |
| Positive | 4 | 12 |  | 2 | 13 |  |
| Negative | 4 | 7 |  | 6 | 5 |  |
| **Progesteron receptor** |  |  | 0,7 |  |  | **0,038** |
| Positiv | 4 | 12 |  | 2 | 13 |  |
| Negativ | 4 | 7 |  | 6 | 5 |  |
| **HER2/neu** |  |  | >0.9 |  |  | >0.9 |
| Positive | 3 | 6 |  | 3 | 6 |  |
| Negative | 5 | 13 |  | 5 | 12 |  |
| **nCTX** |  |  | >0.9 |  |  | 0,7 |
| Yes | 3 | 7 |  | 2 | 7 |  |
| No | 5 | 12 |  | 6 | 11 |  |

**Supplement 1C**: Results of the Fisher´s exact test for association between clinicopathological features of primary breast cancer and expression of CD86. TN: tumor nest; TS: tumor stroma; nCTX: neoadjuvant chemotherapy; n.d.: not determinable.

|  | **CD86, TN** | | | **CD86, TS** | | |
| --- | --- | --- | --- | --- | --- | --- |
|  | **High,**  **n = 11** | **Low,**  **n = 16** | **p** | **High,**  **n = 9** | **Low,**  **n = 17** | **p** |
| **Tumor size** |  |  | 0,9 |  |  | 0,4 |
| 1 | 2 | 5 |  | 2 | 5 |  |
| 2 | 4 | 5 |  | 2 | 7 |  |
| 3 | 2 | 2 |  | 3 | 1 |  |
| 4 | 2 | 3 |  | 1 | 3 |  |
| n.d. | 1 | 1 |  | 1 | 1 |  |
| **Node status** |  |  | 0,4 |  |  | 0,2 |
| N0 | 2 | 6 |  | 1 | 7 |  |
| N+ | 9 | 10 |  | 8 | 10 |  |
| **Metastasic status** |  |  | 0,4 |  |  | >0.9 |
| M0 | 8 | 9 |  | 6 | 11 |  |
| M+ | 3 | 7 |  | 3 | 6 |  |
| **Grading** |  |  | **0,018** |  |  | 0,1 |
| 2 | 2 | 11 |  | 2 | 11 |  |
| 3 | 9 | 5 |  | 7 | 6 |  |
| **Estrogen receptor** |  |  | 0,7 |  |  | 0,1 |
| Positive | 6 | 10 |  | 3 | 12 |  |
| Negative | 5 | 6 |  | 6 | 5 |  |
| **Progesteron receptor** |  |  | 0,7 |  |  | 0,1 |
| Positiv | 6 | 10 |  | 3 | 12 |  |
| Negativ | 5 | 6 |  | 6 | 5 |  |
| **HER2/neu** |  |  | 0,1 |  |  | 0,7 |
| Positive | 6 | 3 |  | 4 | 5 |  |
| Negative | 5 | 13 |  | 5 | 12 |  |
| **nCTX** |  |  | 0,7 |  |  | 0,4 |
| Yes | 5 | 5 |  | 2 | 7 |  |
| No | 6 | 11 |  | 7 | 10 |  |

**Supplement 1D**: Results of the Fisher´s exact test for association between clinicopathological features of primary breast cancer and expression of PD-L1. TN: tumor nest; TS: tumor stroma; nCTX: neoadjuvant chemotherapy; n.d.: not determinable.

| **Klinische Parameter** | **PD-L1** | | |
| --- | --- | --- | --- |
|  | **Positive,**  **n = 11** | **Negative,**  **n = 16** | **p** |
| **Tumor size** |  |  | 0,7 |
| 1 | 2 | 5 |  |
| 2 | 5 | 4 |  |
| 3 | 1 | 3 |  |
| 4 | 2 | 3 |  |
| n.d. | 1 | 1 |  |
| **Node status** |  |  | 0,4 |
| N0 | 2 | 6 |  |
| N+ | 9 | 10 |  |
| **Metastasic status** |  |  | >0.9 |
| M0 | 7 | 10 |  |
| M+ | 4 | 6 |  |
| **Grading** |  |  | 0,7 |
| 2 | 6 | 7 |  |
| 3 | 5 | 9 |  |
| **Estrogen receptor** |  |  | 0,3 |
| Positive | 5 | 11 |  |
| Negative | 6 | 5 |  |
| **Progesteron receptor** |  |  | 0,3 |
| Positiv | 5 | 11 |  |
| Negativ | 6 | 5 |  |
| **HER2/neu** |  |  | 0,4 |
| Positive | 5 | 4 |  |
| Negative | 6 | 12 |  |
| **nCTX** |  |  | 0,7 |
| Yes | 5 | 5 |  |
| No | 6 | 11 |  |

**Supplement 2A**: Results of the Fisher´s exact test for association between clinicopathological features of brain metastases and expression of CD68. TN: tumor nest; TS: tumor stroma; BM: brain metastases; n.d.: not determinable.

|  | **CD68, TN** | | | **CD68, TS** | | |
| --- | --- | --- | --- | --- | --- | --- |
|  | **High,**  **n = 22** | **Low,**  **n = 31** | **p** | **High,**  **n = 20** | **Low,**  **n = 24** | **p** |
| **Meningeal carcinomatosis** |  |  | **0,016** |  |  | 0,7 |
| Yes | 0 | 8 |  | 2 | 4 |  |
| No | 22 | 22 |  | 17 | 20 |  |
| n.d. | 0 | 1 |  | 1 | 0 |  |
| **Dexamethason treatment** |  |  | 0,8 |  |  | 0,4 |
| Yes | 11 | 17 |  | 9 | 15 |  |
| No | 11 | 13 |  | 11 | 9 |  |
| n.d. | 0 | 1 |  |  |  |  |
| **Number of BM** |  |  | 0,6 |  |  | 0,8 |
| solitary | 11 | 12 |  | 7 | 10 |  |
| multiple | 11 | 18 |  | 13 | 14 |  |
| n.d. | 0 | 1 |  |  |  |  |
| **Presentation** |  |  | 0,6 |  |  | 0,6 |
| synchronous | 1 | 3 |  | 1 | 3 |  |
| metachronous | 21 | 28 |  | 19 | 21 |  |
| **Cerebral relaps** |  |  | 0,5 |  |  | 0,7 |
| Yes | 4 | 9 |  | 5 | 4 |  |
| No | 18 | 22 |  | 15 | 20 |  |

**Supplement 2B**: Results of the Fisher´s exact test for association between clinicopathological features of brain metastases and expression of CD163. TN: tumor nest; TS: tumor stroma; BM: brain metastases; n.d.: not determinable.

|  | **TN** | | | **TS** | | |
| --- | --- | --- | --- | --- | --- | --- |
|  | **High,**  **n = 17** | **Low,**  **n = 35** | **p** | **High,**  **n = 20** | **Low,**  **n = 24** | **p** |
| **Meningeal carcinomatosis** |  |  | **0,04** |  |  | 0,7 |
| Yes | 0 | 8 |  | 2 | 4 |  |
| No | 17 | 26 |  | 18 | 19 |  |
| n.d. | 0 | 1 |  | 0 | 1 |  |
| **Dexamethason treatment** |  |  | 0,8 |  |  | 0,2 |
| Yes | 10 | 18 |  | 13 | 11 |  |
| No | 7 | 17 |  | 7 | 13 |  |
| n.d. | 0 | 1 |  |  |  |  |
| **Number of BM** |  |  | 0,4 |  |  | 0,4 |
| solitary | 9 | 13 |  | 6 | 11 |  |
| multiple | 8 | 21 |  | 14 | 13 |  |
| n.d. | 0 | 1 |  |  |  |  |
| **Presentation** |  |  | 0,3 |  |  | 0,11 |
| synchronous | 0 | 4 |  | 0 | 4 |  |
| metachronous | 17 | 31 |  | 20 | 20 |  |
| **Cerebral relaps** |  |  | 0,5 |  |  | >0,9 |
| Yes | 3 | 10 |  | 4 | 5 |  |
| No | 14 | 25 |  | 16 | 19 |  |

**Supplement 2C**: Results of the Fisher´s exact test for association between clinicopathological features of brain metastases and expression of CD86. TN: tumor nest; TS: tumor stroma; BM: brain metastases; n.d.: not determinable.

|  | **CD86, TN** | | | **CD86, TS** | | |
| --- | --- | --- | --- | --- | --- | --- |
|  | **High,**  **n = 20** | **Low,**  **n = 32** | **p** | **High,**  **n = 15** | **Low,**  **n = 29** | **p** |
| **Meningeal carcinomatosis** |  |  | 0,13 |  |  | 0,6 |
| Yes | 1 | 7 |  | 1 | 5 |  |
| No | 19 | 24 |  | 13 | 24 |  |
| n.d. | 0 | 1 |  | 1 | 0 |  |
| **Dexamethason treatment** |  |  | 0,4 |  |  | 0,2 |
| Yes | 9 | 19 |  | 6 | 18 |  |
| No | 11 | 13 |  | 9 | 11 |  |
| n.d. |  |  |  |  |  |  |
| **Number of BM** |  |  | 0,6 |  |  | 0,1 |
| solitary | 10 | 12 |  | 3 | 14 |  |
| multiple | 10 | 19 |  | 12 | 15 |  |
| n.d. | 0 | 1 |  |  |  |  |
| **Presentation** |  |  | 0,2 |  |  | 0,3 |
| synchronous | 0 | 4 |  | 0 | 4 |  |
| metachronous | 20 | 28 |  | 15 | 25 |  |
| **Cerebral relaps** |  |  | 0,7 |  |  | >0,9 |
| Yes | 4 | 9 |  | 3 | 6 |  |
| No | 16 | 23 |  | 12 | 23 |  |

**Supplement 2D**: Results of the Fisher´s exact test for association between clinicopathological features of brain metastases and expression of PD-L1. TN: tumor nest; TS: tumor stroma; BM: brain metastases; n.d.: not determinable.

|  | **PD-L1** | | |
| --- | --- | --- | --- |
|  | **Positive,**  **n = 22** | **Negative,**  **n = 31** | **p** |
| **Meningeal carcinomatosis** |  |  | 0,12 |
| Yes | 1  1 | 7 |  |
| No | 21 | 23 |  |
| n.d. | 0 | 1 |  |
| **Dexamethason treatment** |  |  | 0,3 |
| Yes | 14 | 14 |  |
| No | 8 | 16 |  |
| n.d. | 0 | 1 |  |
| **Number of BM** |  |  | 0,6 |
| solitary | 15 | 14 |  |
| multiple | 7 | 16 |  |
| n.d. |  | 1 |  |
| **Presentation** |  |  |  |
| synchronous | 1 | 3 | 0,2 |
| metachronous | 21 | 28 |  |
| **Cerebral relaps** |  |  |  |
| Yes | 7 | 6 | 0,3 |
| No | 15 | 25 |  |
